# Exposure to polycyclic aromatic hydrocarbons during pregnancy and breast tissue composition in adolescent daughters and their mothers: a prospective cohort study

**DOI:** 10.1186/s13058-022-01546-8

**Published:** 2022-07-11

**Authors:** Rebecca D. Kehm, E. Jane Walter, Sabine Oskar, Melissa L. White, Parisa Tehranifar, Julie B. Herbstman, Frederica Perera, Lothar Lilge, Rachel L. Miller, Mary Beth Terry

**Affiliations:** 1 Department of Epidemiology, Mailman School of Public Health, Columbia University, 722 W 168th St, Room 1605, New York, NY 10032 USA; 2 Princess Margaret Cancer Centre, University Health Network, 101 College St, Toronto, ON M5G 0A3 Canada; 3 Herbert Irving Comprehensive Cancer Center, Columbia University Medical Center, New York, NY 10032 USA; 4 Department of Environmental Health Sciences, Mailman School of Public Health, Columbia University, New York, NY 10032 USA; 5 Department of Medical Biophysics, University of Toronto, 101 College St, Toronto, ON M5G 0A3 Canada; 6 Division of Clinical Immunology, Department of Medicine, Icahn School of Medicine at Mount Sinai, 1 Gustave L Levy Place, New York, NY 10029 USA

**Keywords:** Polycyclic aromatic hydrocarbons, Endocrine disrupting chemicals, Tobacco smoke, Breast cancer risk, Breast density, Breast tissue composition

## Abstract

**Background:**

Polycyclic aromatic hydrocarbons (PAH), which are found in air pollution, have carcinogenic and endocrine disrupting properties that might increase breast cancer risk. PAH exposure might be particularly detrimental during pregnancy, as this is a time when the breast tissue of both the mother and daughter is undergoing structural and functional changes. In this study, we tested the hypothesis that ambient PAH exposure during pregnancy is associated with breast tissue composition, measured one to two decades later, in adolescent daughters and their mothers.

**Methods:**

We conducted a prospective analysis using data from a New York City cohort of non-Hispanic Black and Hispanic mother–daughter dyads (recruited 1998–2006). During the third trimester of pregnancy, women wore backpacks containing a continuously operating air sampling pump for two consecutive days that measured ambient exposure to eight carcinogenic higher molecular weight nonvolatile PAH compounds (Σ8 PAH) and pyrene. When daughters (*n* = 186) and mothers (*n* = 175) reached ages 11–20 and 29–55 years, respectively, optical spectroscopy (OS) was used to evaluate measures of breast tissue composition (BTC) that positively (water content, collagen content, optical index) and negatively (lipid content) correlate with mammographic breast density, a recognized risk factor for breast cancer. Multivariable linear regression was used to evaluate associations between ambient PAH exposure and BTC, overall and by exposure to household tobacco smoke during pregnancy (yes/no). Models were adjusted for race/ethnicity, age, and percent body fat at OS.

**Results:**

No overall associations were found between ambient PAH exposure (Σ8 PAH or pyrene) and BTC, but statistically significant additive interactions between Σ8 PAH and household tobacco smoke exposure were identified for water content and optical index in both daughters and mothers (interaction *p* values < 0.05). Σ8 PAH exposure was associated with higher water content (*β*_daughters_ = 0.42, 95% CI = 0.15–0.68; *β*_mothers_ = 0.32, 95% CI = 0.05–0.61) and higher optical index (*β*_daughters_ = 0.38, 95% CI = 0.12–0.64; *β*_mothers_ = 0.38, 95% CI = 0.12–0.65) in those exposed to household tobacco smoke during pregnancy; no associations were found in non-smoking households (interaction *p* values < 0.05).

**Conclusions:**

Exposure to ambient Σ8 PAH and tobacco smoke during pregnancy might interact synergistically to impact BTC in mothers and daughters. If replicated in other cohorts, these findings might have important implications for breast cancer risk across generations.

**Supplementary Information:**

The online version contains supplementary material available at 10.1186/s13058-022-01546-8.

## Background

Polycyclic aromatic hydrocarbons (PAH) are ubiquitous environmental pollutants that are found in several sources, including vehicular emissions, coal-burning plants, tobacco smoke, and the burning of biomass [1]. PAH have both carcinogenic and endocrine disrupting properties (pro- or anti-estrogenic effects depending on the compound) [1–3], and mounting evidence supports that PAH exposure is associated with increased breast cancer risk (as reviewed in [4, 5]). PAH exposure during pregnancy might be particularly detrimental because this is a time when the breast tissue of both the mother and daughter is undergoing structural and functional changes, and is thus potentially more susceptible to environmental carcinogens [6]. This is supported by animal studies that have found pregnancy-related exposure to certain endocrine disrupting chemicals (e.g., dioxin, atrazine, Bisphenol A) alters mammary gland development and morphology in both the mother and offspring [7, 8]. In a recent study, we found that prenatal exposure to ambient PAH altered estrogen receptor α mRNA expression and induced methylation in the estrogen receptor *α* promoter in the mammary tissue of both offspring and grand-offspring mice, as well as functional outcomes such as mammary cell proliferation in offspring mice [9]. Yet, there are currently limited epidemiological data to support the hypothesis that pregnancy-related PAH exposure impacts breast tissue composition (BTC) in either the mother or daughter.

Mammographic breast density (MBD), the amount of collagen, epithelial, and stromal cells relative to fat in the breast, is consistently found to be one of the strongest biomarkers of breast cancer risk in older adult women of mammographic screening age (fourfold to sixfold increase in breast cancer risk for women in the highest versus lowest category of density) [10–13]. Therefore, an association between pregnancy-related PAH exposure and MBD, or other measures of BTC that are associated with MBD, could have important implications for breast cancer risk. While it is not yet known if pregnancy-related PAH exposure impacts BTC in mothers or daughters, there are data supporting that BTC can be modified by environmental chemical exposures. This includes a longitudinal study that found prenatal exposure to dichlorodiphenyltrichloroethane was associated with higher MBD in women, aged 44–54 years with a family history of breast cancer [14]. Cross-sectional studies have found that high versus low urinary concentrations of endocrine disrupting phthalates and phenols are associated with higher MBD in adult women [15], as well as higher percent fibroglandular volume in the breast tissue of adolescent girls [16]. Tobacco smoke exposure has been associated with lower MBD [17–19], possibly due to the anti-estrogenic effects of some chemicals found in tobacco smoke [20]. Studies examining air pollution in association with MBD have produced inconsistent results and were limited by the use of ecological exposure data [21–24]. Further, studies of air pollution and MBD have only been conducted in cohorts of older adult women, likely because mammography is not recommended for routine use until at least age 40 years. In this study, we used prospectively collected data to test the hypothesis that exposure to ambient PAH during pregnancy is associated with BTC, measured by optical spectroscopy (OS) one to two decades later, in adolescent daughters and their mothers.

## Methods

### Study sample

We used data from the Columbia Center for Children’s Environmental Health (CCCEH) birth cohort, which was initiated to study the health effects of air pollution in non-Hispanic Black and Hispanic children living in a low-income area of New York City [25–27]. From 1998 to 2006, women in their third trimester of pregnancy were recruited from prenatal clinics at New York Presbyterian and Harlem Hospitals, as well as satellite clinics. Enrollment was restricted to women who were non-active cigarette smokers in the age range of 18–35 years. Additional details on the cohort, including retention rates, are available elsewhere [25–27]. Beginning in 2016, we enrolled 216 of the 285 (76%) mother–daughter pairs still active in the CCCEH cohort into a follow-up study through the Breast Cancer and the Environment Research Program (BCERP) consortia (Columbia-BCERP Study). Daughters and mothers were ages 11–20 and 29–55 years, respectively, at enrollment into the Columbia-BCERP Study and attended at least one clinic visit at which they completed questionnaires, biospecimen collection, anthropometry measurements, and OS measurement of the breast. For this analysis, we included 182 daughters and 175 mothers with complete data on BTC, ambient PAH exposure (from personal air monitoring), and household smoke exposure (from the baseline questionnaire completed by mothers during pregnancy). Written informed consent was obtained from all mothers for themselves and for their daughters under age 18 years at the clinic visit, along with written informed assent from daughters. Written informed consent was also obtained from daughters who were 18 years or older at enrollment in the Columbia-BCERP Study. This study was approved by the IRB at Columbia University. All methods in this study were conducted in accordance with relevant guidelines and regulations (Declaration of Helsinki).

### Assessment of ambient PAH exposure

During the third trimester of pregnancy, women wore a small backpack containing a personal air quality monitor during the daytime hours for two consecutive days and placed the backpack near their bed at night. The personal air sampling pumps operated continuously over this period, collecting vapors and particles ≤ 2.5 microns in diameter on a pre-cleaned quartz microfiber filter and a pre-cleaned polyurethane foam cartridge backup. The samples were analyzed at Southwest Research Institute (San Antonio, Texas) for pyrene, a lower molecular weight semi-volatile PAH, and eight higher molecular weight nonvolatile carcinogenic PAH compounds including benzo[a]anthracene, chrysene, benzo[b]fluoranthene, benzo[k]fluoranthene, benzo[α]pyrene, indeno[1,2,3-cd]pyrene, dibenz[a,h]anthracene, and benzo[g,h,i]perylene, as previously described [28]. For quality control, each personal monitoring result was assessed for accuracy in flow rate, time, and completeness of documentation. Previous studies using the CCCEH cohort have shown that the 8 higher molecular weight nonvolatile PAH compounds are significantly intercorrelated (*ρ* values ranging from 0.45 to 0.94; all *p* values < 0.001 by Spearman’s rank) [26]. Therefore, we used a summary measure of the 8 higher molecular weight nonvolatile PAH compounds in our analysis (hereafter referred to as Σ8 PAH). We analyzed pyrene separately because it is a semi-volatile PAH compound with markedly higher exposure levels in our sample compared with the individual nonvolatile PAH compounds. We evaluated Σ8 PAH and pyrene exposure as continuous variables (log-transformed and standardized to a mean of zero and standard deviation of one) and categorized into tertiles based on the sample distribution.

### Household tobacco smoke exposure during pregnancy

On the baseline questionnaire, which was administered during the third trimester of pregnancy, mothers reported if there were any tobacco smokers in the household during pregnancy (yes vs. no). This self-reported measure was previously found to be highly correlated with plasma cotinine levels measured in cord blood (chi-square tests for trend *p* < 0.001) [29].

### Measurement of BTC by OS

We used OS to obtain measures of BTC in adolescent daughters and their mothers. OS is a non-imaging and minimally invasive (does not involve ionizing radiation or breast compression) method that quantifies the biochemical composition of the breast tissue by capturing the red and near-infrared light absorption by chromophores in breast tissue in the 650 to 1060 nm spectral range. For this analysis, we focused on the three chromophores that most strongly correlate with MBD. This includes water content and collagen content, which positively correlate with MBD, and lipid content, which negatively correlates with MBD [30]. We also calculated a combined optical index, defined as [((collagen + water)*scattering power)/ lipid], because previous studies have found that MBD is more strongly correlated with this composite measure than with the individual chromophore measures (e.g., water content) [31].

Two versions of the OS device were used during the study period, including the LEGACY OS device (59% of the sample) and the CUPS OS device (41% of the sample). The design and methodology of these device have been previously described in detail (see [32]). Instrument throughput was quantified using a standard silicone reference before and after each participant’s breast measurement. Up to 12 and 24 spectra were collected per breast for the CUPS OS device and LEGACY OS device, respectively. These spectra were collected for multiple source–detector distances placed at different locations, thus optically interrogating different, but overlapping tissue volumes. A fitting algorithm was developed to determine the concentrations of the five dominant chromophores and the two light scattering parameters of breast tissue. For the fitting algorithm, simulated spectra were calculated using the known absorption spectra of the tissue chromophores and light scattering properties and look-up tables of expected detector signal values for different tissue absorption and scattering properties, generated using a Monte Carlo simulation-based light propagation program (FullMonte) [33]. The fit parameters were constrained to within expected ranges based on previous studies with generous latitude to allow for population differences and ensure physiologically reasonable values [30]. The concentrations of water, lipid, and collagen were constrained to 4–90%, 10–95%, and 1–30%, respectively. Fitting was performed in MATLAB (The MathWorks Inc., Natick, MA, USA).

Only spectra with data for at least 7 wavelengths, including at least 985 nm and 905 nm or 940 nm (distinguishing the primary lipid and water absorption peaks), were considered for subsequent data analysis. Chromophore fitting was performed for multiple starting points, and the best fit was selected based on the lowest chi-squared difference between the measured and fitted spectra, with the additional constraint that no more than two parameters reached a predetermined minima or maxima. We averaged chromophore and light scattering data over all evaluable spectra in both breasts to generate a single set of chromophore concentrations and light scattering properties for each participant. This is supported by research that has demonstrated symmetry in the optical spectra between breasts in the absence of breast disease [34]. Associations did not statistically significantly differ by OS device type, which was evaluated by including cross-product terms between OS device type (CUPS versus LEGACY) and PAH in models, and so we combined data from the two devices in analyses.

### Statistical analysis

We used multivariable linear regression models to evaluate associations of ambient PAH exposure (Σ8 PAH and pyrene) with BTC (water, lipid, collagen, and optical index) in daughters and mothers, analyzed separately. The measures of BTC were modeled as continuous variables that were log-transformed and standardized to a mean of zero and standard deviation of one. Models were adjusted for race/ethnicity, and age and percent body fat (measured by bioimpedance using an Omron Handheld HBF-360C) at OS measurement. Quadratic and cubic terms for age at OS measurement were included in models for daughters to account for the nonlinear relationship between age and the measures of BTC. These polynomial terms were not statistically significant in models predicting measures of BTC in mothers. We also evaluated models adjusted for self-reported days since last menstruation, maternal education during pregnancy, public assistance during pregnancy, mother’s pre-pregnancy body mass index (BMI), mother’s weight gain during pregnancy, Tanner Stage at OS measurement (daughters only), and age at menarche (daughters only). These covariates did not markedly change associations and so were not included in the final parsimonious models. We tested for additive interactions between the PAH measures and household smoke exposure during pregnancy by including cross-product terms in models and evaluating the Wald test statistic. In daughters, we also tested for interactions by birthweight and childhood BMI (measured by research staff at age 9 years, see [35] for details) based on our previous research supporting that childhood growth modifies associations of environmental exposures with pubertal timing [36, 37]. Statistical tests were two-sided, and p values < 0.05 were considered statistically significant. Analyses were performed using Stata 15.1 (College Station, Texas) [38].

## Results

### Sample characteristics

Mean age at OS measurement was 16.1 years (standard deviation (SD) = 2.4) for daughters and 42.1 years (SD = 5.5) for mothers. Mean water, lipid, and collagen content in the breast tissue was 19.6% (SD = 6.8), 38.3% (SD = 13.9), and 25.2% (SD = 6.6), respectively, in daughters, and 16.0% (SD = 6.5), 43.4% (SD = 11.5), and 24.4% (SD = 6.4), respectively, in mothers. The four measures of BTC were shown to vary by age at OS measurement in mothers and by ages at OS measurement and menarche in daughters (see Additional file 1). Mean level of Σ8 PAH exposure did not statistically significantly differ between smoking versus non-smoking households (3.03 ± 2.77 ng/m^3^ vs. 2.63 ± 1.60 ng/m^3^; *t* test *p* value = 0.27). Additional descriptive characteristics of the study sample by level of Σ8 PAH exposure and household smoke exposure are provided in Table 1.

**Table 1:** Characteristics of daughters and mothers in the Columbia Breast Cancer and the Environment Research Program (Columbia-BCERP) Study by Σ8 PAH and household tobacco smoke exposure during pregnancy

Characteristic	Tertile of Σ8 PAH exposure during pregnancy			*p* value^a^	Tobacco smoker in the household during pregnancy		*p* value^a^
	Low, < 1.75 ng/m^3^	Medium, 1.75 to < 2.95 ng/m^3^	High, ≥ 2.95 ng/m^3^		No	Yes	
*Daughters (n* = *182)*							
Age at OS, years, mean (SD)	15.4 (2.5)	16.3 (2.4)	16.8 (2.1)	0.004	16.0 (2.3)	16.4 (2.5)	0.32
Tanner stage at OS, n (%)				0.05			0.58
Stage 1 or 2	19 (30.7)	5 (8.3)	10 (16.7)		26 (21.5)	8 (13.1)	
Stage 3	18 (29.0)	19 (31.7)	18 (30.0)		36 (29.8)	19 (31.2)	
Stage 4 or 5	22 (35.5)	35 (58.3)	30 (50.0)		55 (45.5)	32 (52.5)	
Unknown	3 (4.8)	1 (1.7)	2 (3.3)		4 (3.3)	2 (3.3)	
Birthweight, grams, mean (SD)	3286 (431)	3380 (458)	3304 (531)	0.51	3376 (455)	3219 (494)	0.04
BMI at age 9, kg/m^2^, mean (SD)	19.6 (4.4)	20.6 (5.2)	19.8 (4.4)	0.54	19.8 (4.9)	20.3 (4.2)	0.50
BMI at OS, kg/m^2^, mean (SD)	24.5 (6.5)	26.7 (7.2)	26.0 (7.6)	0.22	25.5 (7.1)	26.2 (7.2)	0.52
Body fat at OS, %, mean (SD)	29.4 (7.4)	30.7 (8.3)	29.8 (8.3)	0.67	29.8 (8.0)	30.3 (7.8)	0.65
Age at menarche, years, mean (SD)	11.3 (1.4)	11.7 (1.3)	11.7 (1.6)	0.15	11.6 (1.5)	11.5 (1.4)	0.66
Smoker in household during pregnancy, n (%)				0.84			
Yes	19 (30.7)	21 (35.0)	21 (35.0)				
No	43 (69.4)	39 (65.0)	39 (65.0)				
Pyrene exposure during pregnancy, ng/m^3^, mean (SD)	2.9 (2.1)	2.9 (1.3)	3.9 (2.3)	0.007	3.2 (2.1)	3.2 (1.7)	0.81
*Mothers (n* = *175)*							
Age at OS, years, mean (SD)	41.1 (5.8)	42.4 (5.2)	42.9 (5.3)	0.20	42.6 (5.2)	41.1 (5.8)	0.08
Race and ethnicity, n (%)				0.66			0.001
Non-Hispanic Black	20 (33.9)	22 (38.6)	18 (30.5)		30 (25.6)	30 (51.7)	
Hispanic	39 (66.1)	35 (61.4)	41 (69.5)		87 (74.4)	28 (48.3)	
Education during pregnancy, n (%)				0.70			0.95
High school degree or less	44 (74.6)	43 (76.8)	47 (81.0)		90 (77.6)	44 (77.2)	
More than high school	15 (25.4)	13 (23.2)	11 (19.0)		26 (22.4)	13 (22.8)	
Public assistance during pregnancy, n (%)				0.98			0.28
Yes	30 (50.9)	29 (50.9)	28 (49.1)		55 (47.4)	32 (56.1)	
No	29 (49.2)	28 (49.1)	29 (50.9)		61 (52.6)	25 (43.9)	
Pre-pregnancy BMI, kg/m^2^, mean (SD)	24.6 (4.5)	26.9 (7.5)	25.9 (4.9)	0.12	25.5 (5.4)	26.4 (6.6)	0.36
Weight gain during pregnancy, kg, mean (SD)	13.4 (7.9)	12.0 (6.3)	11.9 (6.5)	0.42	11.9 (6.6)	13.5 (7.3)	0.14
BMI at OS, kg/m^2^, mean (SD)	29.9 (6.5)	31.7 (7.7)	30.7 (6.9)	0.41	30.3 (6.9)	31.6 (7.2)	0.24
Body fat at OS, %, mean (SD)	36.2 (7.6)	37.2 (6.0)	36.7 (7.0)	0.77	36.2 (6.7)	37.6 (7.2)	0.24
Tobacco smoker in the household during pregnancy, n (%)				0.86			
Yes	18 (30.5)	20 (35.1)	20 (33.9)				
No	41 (69.5)	37 (64.9)	39 (66.1)				
Pyrene exposure during pregnancy, ng/m^3^, mean (SD)	2.9 (2.2)	2.8 (1.2)	3.9 (2.3)	0.006	3.3 (2.1)	3.1 (1.7)	0.61

*BMI* Body mass index; *OS* Optical index; *PAH* Polycyclic aromatic hydrocarbons; *SD* standard deviation

^a^*P* value reported from Pearson chi-squared test for categorical variables and either the two-sample test or analysis of variance test for continuous variables

### Prenatal exposure to ambient PAH and BTC in daughters

**Fig. 1:**
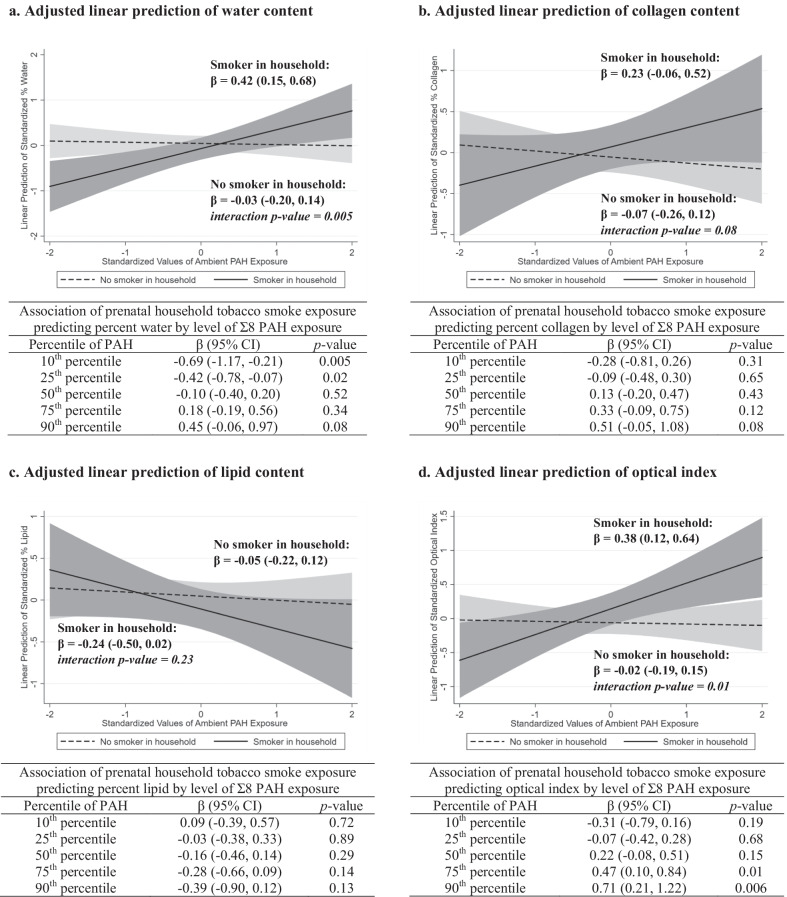
Beta coefficients and 95% confidence intervals are estimated from linear regression models with a cross-product term between log-transformed Σ8 PAH (modeled as a continuous variable) and household tobacco smoke exposure during pregnancy (any/none) and adjustment for race/ethnicity, age (linear, cubic, and quadratic terms) and percent body fat at optical spectroscopy

As shown in Table 2, there were no overall associations between prenatal exposure to ambient PAH (Σ8 PAH and pyrene modeled as continuous variables) or household smoke and the measures of BTC in daughters. No associations were found when the PAH measures were modeled as categorical variables (data not shown). However, statistically significant additive interactions between prenatal exposure to Σ8 PAH (modeled continuously) and household tobacco smoke were found predicting water content (interaction *p* value = 0.005) and optical index (interaction *p* value = 0.01) in the breast tissue of daughters. In daughters with prenatal household smoke exposure, Σ8 PAH was statistically significantly associated with higher water content (*β* = 0.42, 95% CI = 0.15 to 0.68) and higher optical index (*β* = 0.38, 95% CI = 0.12 to 0.64), but no associations were found in daughters born in non-smoking households (Fig. 1). Household tobacco smoke exposure was statistically significantly associated with lower water content in girls with lower PAH exposure (e.g., Σ8 PAH = 10th percentile: β = − 0.69, 95% CI = − 1.17 to − 0.21), but not in girls with higher PAH exposure. A similar, although not statistically significant, additive interaction between Σ8 PAH and household smoke exposure was found predicting collagen content in daughters (interaction p value = 0.08). No statistically significant additive interactions were found between prenatal exposure to pyrene and household smoke predicting the BTC measures in daughters (data not shown).

**Table 2:** Adjusted linear associations of prenatal exposure to ambient PAH and measures of breast tissue composition in adolescent daughters, ages 11–20 years, from the Columbia Breast Cancer and the Environment Research Program Columbia-BCERP) Study; *N* = 182

Model covariate	Water content		Lipid content		Collagen content		Optical index	
	*β* (95% CI)	*p* value	*β* (95% CI)	*p* value	*β* (95% CI)	*p* value	*β* (95% CI)	*p* value
*Prenatal exposures*								
Σ8 PAH^a^	0.10 (− 0.04, 0.25)	0.17	− 0.10 (− 0.24, 0.04)	0.17	0.01 (− 0.15, 0.18)	0.86	0.09 (− 0.05, 0.24)	0.20
Pyrene^b^	0.01 (− 0.13, 0.16)	0.88	− 0.03 (− 0.17, 0.11)	0.69	− 0.01 (− 0.16, 0.15)	0.94	0.03 (− 0.11, 0.18)	0.63
Tobacco smoke^c^	− 0.15 (− 0.46, 0.16)	0.33	− 0.14 (− 0.43, 0.16)	0.37	0.10 (− 0.23, 0.43)	0.56	0.17 (− 0.13, 0.47)	0.27
*Covariates*								
Age, linear term^d^	− 12.53 (− 19.51, − 5.55)	0.001	10.94 (4.16, 17.71)	0.002	0.00 (− 7.60, 7.59)	0.99	− 8.83 (− 15.65, − 2.01)	0.01
Age, quadratic term^d^	0.81 (0.38, 1.25)	< 0.001	− 0.71 (− 1.13, − 0.28)	0.001	− 0.01 (− 0.48, 0.46)	0.97	0.57 (0.14, 0.99)	0.009
Age, cubic term^d^	− 0.02 (− 0.03, − 0.01)	< 0.001	0.02 (0.01, 0.02)	0.001	0.00 (− 0.01, 0.01)	0.93	− 0.01 (− 0.02, 0.00)	0.007
Race/ethnicity^e^	− 0.27 (− 0.57, 0.02)	0.07	0.61 (0.32, 0.90)	< 0.001	− 0.30 (− 0.63, 0.02)	0.06	− 0.75 (− 1.04, − 0.46)	< 0.001
Percent body fat^f^	− 0.23 (− 0.40, − 0.05)	0.01	0.32 (0.15, 0.49)	< 0.001	− 0.11 (− 0.30, 0.09)	0.28	− 0.16 (− 0.33, 0.01)	0.07

^a^Modeled as a one-standard deviation change in log-transformed Σ8 polycyclic aromatic hydrocarbons (PAH) exposure; adjusted for race/ethnicity, age at optical spectroscopy, and percent body fat at optical spectroscopy

^b^Modeled as a one-standard deviation change in log-transformed pyrene exposure; adjusted for race/ethnicity, age at optical spectroscopy, and percent body fat at optical spectroscopy

^c^Modeled as any tobacco smoker in the household during pregnancy vs. none; adjusted for race/ethnicity, age at optical spectroscopy, and percent body fat at optical spectroscopy

^d^Modeled as a one-year change in age; adjusted for Σ8 PAH exposure during pregnancy, race/ethnicity, and percent body fat at optical spectroscopy

^e^Modeled as Hispanic versus non-Hispanic Black; adjusted for Σ8 PAH exposure during pregnancy, age at optical spectroscopy, and percent body fat at optical spectroscopy

^f^Modeled as a 10% change in body fat; adjusted for Σ8 PAH exposure during pregnancy, race/ethnicity, and age at optical spectroscopy

**Fig. 2:**
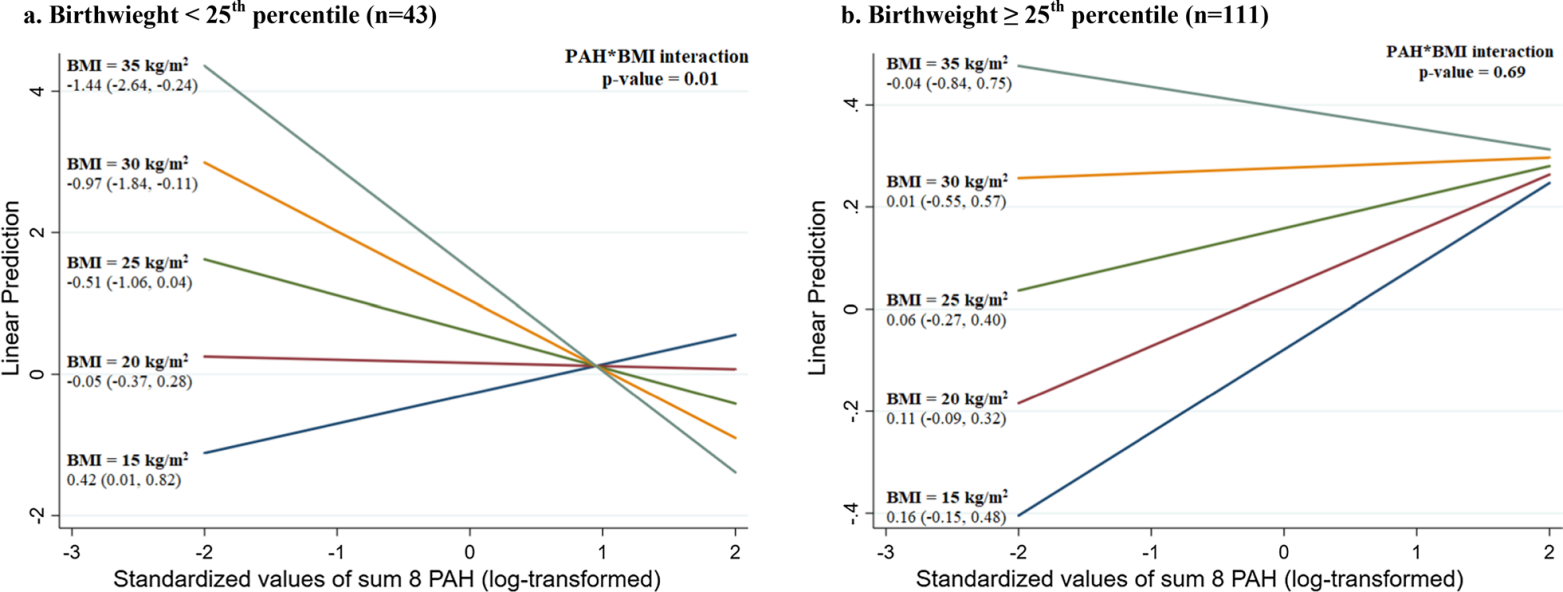
Beta coefficients and 95% confidence intervals are estimated from linear regression models with a cross-product term between log-transformed Σ8 PAH (modeled as a continuous variable) and childhood body mass index (BMI) measured at age 9 years (modeled as a continuous variable. Models are stratified by birthweight percentile (< 25th percentile vs. ≥ 25th percentile based on the Centers for Disease Control growth charts) and adjusted for race/ethnicity, age (linear, cubic, and quadratic terms) and percent body fat at optical spectroscopy

As shown in Fig. 2, in daughters with a birthweight below the 25th percentile based on the Centers for Disease Control growth charts (*n* = 43) [39], prenatal exposure to Σ8 PAH was positively associated with water content in those with lower childhood BMI measured at age 9 years (e.g., BMI = 15 kg/m^2^: *β* = 0.42, 95%CI = 0.01 to 0.82) and negatively associated with water content in those with higher childhood BMI (e.g., BMI = 30 kg/m^2^: *β* = − 0.97, 95%CI = − 1.84 to − 0.11; PAH*BMI interaction *p* value = 0.01). However, no interaction between Σ8 PAH and childhood BMI was found in the subgroup of daughters with a birthweight at or above the 25th percentile (*n* = 111; PAH*BMI interaction *p* value = 0.69). The statistically significant additive interaction between Σ8 PAH exposure and childhood BMI in daughters with a birthweight below the 25th percentile was still seen when the sample was restricted to daughters born in smoking households (data not shown).

### Pregnancy exposure to ambient PAH and BTC in mothers

**Fig. 3:**
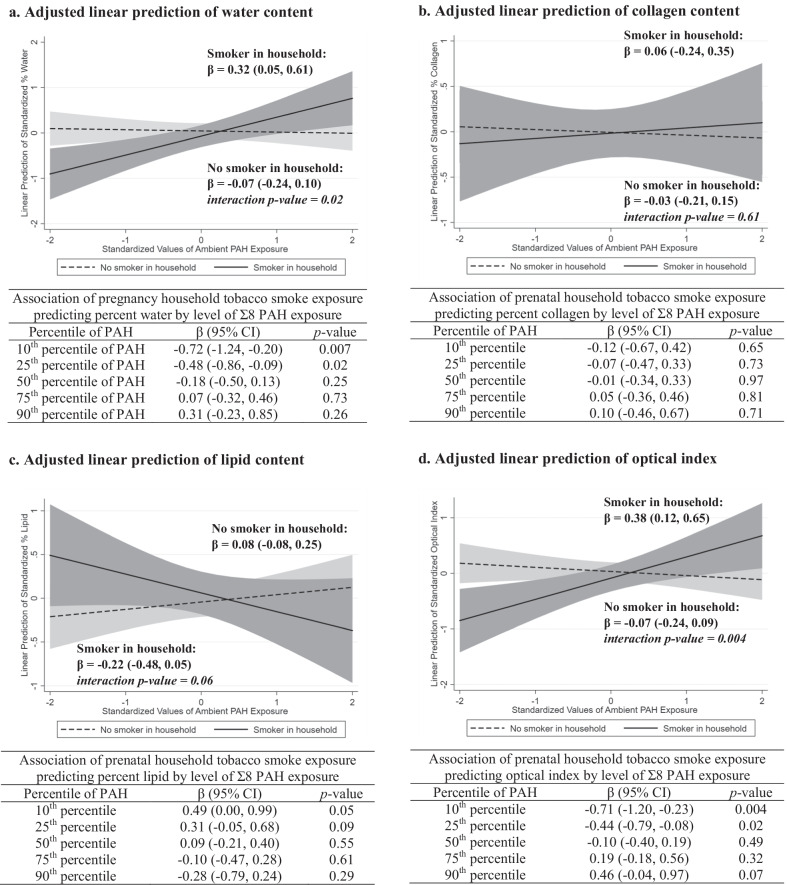
Beta coefficients and 95% confidence intervals are estimated from linear regression models with a cross-product term between log-transformed Σ8 PAH (modeled as a continuous variable) and household tobacco smoke exposure during pregnancy (any/none) and adjustment for race/ethnicity, age, and percent body fat at optical spectroscopy

No overall associations were found between exposure to ambient Σ8 PAH, pyrene, or household smoke during pregnancy and the measures of BTC in mothers (Table 3). Similar to what was found in daughters, statistically significant additive interactions between Σ8 PAH and household smoke exposure during pregnancy were found predicting water content (PAH*smoke interaction *p* value = 0.02) and optical index (PAH*smoke interaction *p* value = 0.004) in mothers (Fig. 3). However, unlike in daughters, there was no suggestion of an additive interaction between Σ8 PAH and household smoke exposure predicting collagen content in mothers (interaction *p* value = 0.61).

**Table 3:** Adjusted linear associations of pregnancy-related exposure to ambient PAH and measures of breast tissue composition in mothers, ages 29–55 years, from the Columbia Breast Cancer and the Environment Research Program Columbia-BCERP) Study; *N* = 175

Model covariate	Water content		Lipid content		Collagen content		Optical index	
	*β* (95% CI)	*p* value	*β* (95% CI)	*p* value	*β* (95% CI)	*p* value	*β* (95% CI)	*p* value
*Pregnancy exposures*								
Σ8 PAH^a^	0.03 (− 0.11, 0.18)	0.65	0.00 (− 0.14, 0.15)	0.96	− 0.01 (− 0.16, 0.15)	0.93	0.05 (− 0.09, 0.19)	0.50
Pyrene^b^	− 0.03 (− 0.18, 0.12)	0.70	0.04 (− 0.10, 0.18)	0.56	− 0.11 (− 0.26, 0.05)	0.17	− 0.05 (− 0.19, 0.09)	0.46
Tobacco smoke^c^	− 0.22 (− 0.54, 0.10)	0.19	0.12 (− 0.19, 0.42)	0.45	− 0.01 (− 0.34, 0.32)	0.94	− 0.14 (− 0.44, 0.16)	0.37
*Covariates*								
Age, linear term^d^	− 0.05 (− 0.08, − 0.02)	0.001	0.04 (0.02, 0.07)	0.002	− 0.02 (− 0.05, 0.01)	0.16	− 0.05 (− 0.08, − 0.02)	< 0.001
Race/ethnicity^e^	0.17 (− 0.14, 0.47)	0.28	0.55 (0.26, 0.84)	< 0.001	− 0.39 (− 0.71, − 0.08)	0.01	− 0.66 (− 0.95, − 0.37)	< 0.001
Percent body fat^f^	− 0.11 (− 0.32, 0.11)	0.34	0.32 (0.11, 0.53)	0.003	− 0.24 (− 0.47, − 0.02)	0.04	− 0.18 (− 0.39, 0.03)	0.09

^a^Modeled as a one-standard deviation change in log-transformed Σ8 polycyclic aromatic hydrocarbons (PAH) exposure; adjusted for race/ethnicity, age at optical spectroscopy, and percent body fat at optical spectroscopy

^b^Modeled as a one-standard deviation change in log-transformed pyrene exposure; adjusted for race/ethnicity, age at optical spectroscopy, and percent body fat at optical spectroscopy

^c^Modeled as any tobacco smoker in the household during pregnancy vs. none; adjusted for race/ethnicity, age at optical spectroscopy, and percent body fat at optical spectroscopy

^d^Modeled as a one-year change in age; adjusted for Σ8 PAH exposure during pregnancy, race/ethnicity, and percent body fat at optical spectroscopy

^e^Modeled as Hispanic versus non-Hispanic Black; adjusted for Σ8 PAH exposure during pregnancy, age at optical spectroscopy, and percent body fat at optical spectroscopy

^f^Modeled as a 10% change in body fat; adjusted for Σ8 PAH exposure during pregnancy, race/ethnicity, and age at optical spectroscopy

## Discussion

In this prospective cohort study, we found limited evidence of an overall association between pregnancy-related exposure to ambient PAH (Σ8 PAH and pyrene) and BTC in adolescent daughters and their mothers. However, when we stratified by household smoke exposure status during pregnancy, we found that ambient Σ8 PAH (but not pyrene) was associated with higher breast density as indicated by higher water content and optical index in both daughters and mothers, as well as possibly higher collagen content in daughters, in those exposed to household smoke during pregnancy. Ambient Σ8 PAH exposure was not associated with BTC in daughters or mothers in non-smoking households during pregnancy. These findings are biologically plausible given that tobacco smoke contains a number of chemicals, in addition to PAH, that could interact synergistically with PAH to alter BTC. Previous studies conducted in the CCCEH cohort have also found synergistic interactions between prenatal exposure to ambient Σ8 PAH, but not pyrene, and environmental tobacco smoke predicting asthma and other respiratory symptoms in children [29, 40], although the biological mechanisms through which environmental chemicals interact to impact respiratory outcomes and BTC might differ. The interaction between Σ8 PAH and environmental tobacco smoke observed for BTC might be due, at least in part, to the impact of tobacco smoke on body size across the life course [41–44], especially given our findings related to Σ8 PAH, birthweight, and childhood BMI (described below). However, mechanistic studies and larger cohort studies are needed to explore these relationships further.

As with ambient PAH exposure, we found no overall associations between household tobacco smoke exposure during pregnancy and BTC in daughters or mothers. However, in those with low levels of Σ8 PAH exposure during pregnancy, household tobacco smoke exposure was associated with indicators of lower breast density in daughters (lower water content) and mothers (higher lipid content and lower water content and optical index), which is consistent with findings from a previous study in which we found that prenatal exposure to maternal tobacco smoking was associated with lower MBD in adult women (aged 39–49 years) [45]. Studies that evaluated contemporary measures of tobacco smoking status and MBD in adult women have also mostly, but not always [46], found a negative association between active smoking and MBD [17–19]. Our findings suggest that this negative association between tobacco smoke exposure and breast density might only occur in the absence of other environmental chemical exposures, such as PAH.

We also found a statistically significant additive interaction between prenatal Σ8 PAH exposure and childhood BMI (measured at age 9 years) predicting water content in the breast tissue of daughters with a birthweight below the 25th percentile, such that prenatal Σ8 PAH exposure was positively associated with water content in those with lower childhood BMI and negatively associated with water content in those with higher childhood BMI. This additive interaction was not found in girls with a birthweight at or above the 25th percentile. Interestingly, in our previous analysis of pubertal timing in the Columbia-BCERP Study, we found that prenatal exposure to Σ8 PAH was associated with later age at menarche in girls with a birthweight below the 25th percentile, but not in girls with a birthweight at or above the 25th percentile [36]. Therefore, it is possible that the association between prenatal Σ8 PAH exposure and BTC is mediated by the timing of menarche, especially given that average water content varied by age at menarche within age-groups in our sample. However, because we did not have repeated measures of BTC in this study, further prospective studies are needed to better understand the relationship between prenatal PAH exposure, pubertal timing, and change in BTC over time in adolescent girls.

This study has several strengths including the use of prospectively collected data from a cohort of mothers and daughters followed since pregnancy. We used personal air monitoring data to measure ambient PAH exposure levels and accounted for household smoke exposure, which is another source of exposure to PAH and other environmental chemicals. Further, we used OS to measure BTC in adolescent daughters and mothers, which is a minimally invasive method for obtaining data on individual breast tissue chromophores that is shown to accurately identify women with high MBD (> 75%) and objectively identify breast Tanner Stage [47, 48]. Limitations of this study include that personal air monitoring was only conducted over a 48-h period during the third trimester of pregnancy, and this short period of exposure assessment may not be representative of long-term exposures throughout pregnancy. However, an indoor air monitoring sub-study conducted in the CCCEH cohort showed that residential indoor PAH exposure levels were fairly stable during the last 6–8 weeks of pregnancy, and home indoor PAH levels were found to be correlated with those in the personal air monitoring samples [49]. We also did not measure exposure to PAH at later time points after pregnancy (e.g., during puberty for daughters or during perimenopause for mothers), which could theoretically be correlated with exposure levels during pregnancy or wash out some of the effects from pregnancy. Further, we did not have repeated measures of BTC to evaluate change over time, which will be important to evaluate in future studies given that BTC is dynamic over the life course. Finally, we did not adjust for multiple comparisons and thus some of the statistically significant findings could be due to chance. However, the consistency in findings across the BTC measures in daughters and mothers (e.g., Σ8 PAH consistently positively associated with indicators of higher breast density in smoking households) gives weight to our findings.

## Conclusions

In conclusion, this study suggests that exposure to PAH and environmental tobacco smoke during pregnancy might interact synergistically to impact BTC in both mothers and daughters. Given that our sample was restricted to non-smoking mothers, these findings suggest that exposures external to the mother, such as paternal smoking, contribute to BTC in daughters. Therefore, more research is needed on the role of paternal factors in breast cancer risk, especially given that most life course studies have predominantly focused on maternal factors. If replicated in other cohorts, our study findings might have important implications for the role of pregnancy-related environmental exposures in breast cancer risk across generations.

## Supplementary Information

**Additional file 1.** Mean predictions of breast tissue chromophores measured from optical spectroscopy (OS) by age at OS measurement quintiles in adolescent daughters and mothers in the Columbia-BCERP Study. Presents the mean water content, collagen content, and lipid content in the breast tissue of daughters and mothers by quintiles of age at optical spectroscopy measurement and age at menarche (daughters only).

## Data Availability

The dataset used and analyzed during this study is available from the corresponding author on reasonable request.

## Abbreviations

BCERP: Breast Cancer and the Environment Research Program
BMI: Body mass index
BTC: Breast tissue composition
CCCEH: Columbia Center for Children’s Environmental Health
MBD: Mammographic breast density
PAH: Polycyclic aromatic hydrocarbons
OS: Optical spectroscopy
SD: Standard deviation
