# Supplementary material for: Exposure to polycyclic aromatic hydrocarbons during pregnancy and breast tissue composition in adolescent daughters and their mothers: a prospective cohort study
Source: Breast Cancer Res. 2022 Jul 11;24:47. doi: 10.1186/s13058-022-01546-8 (PMC9277813; doi:10.1186/s13058-022-01546-8)
Supplement: Supplementary file 1 — Additional file 1. Mean predictions of breast tissue chromophores measured from optical spectroscopy (OS) by age at OS measurement quintiles in adolescent daughters and mothers in the Columbia-BCERP Study. Presents the mean water content, collagen content, and lipid content in the breast tissue of daughters and mothers by quintiles of age at optical spectroscopy measurement and age at menarche (daughters only). [file 13058_2022_1546_MOESM1_ESM.docx]

**Additional File 1.** Mean predictions of breast tissue chromophores measured from optical spectroscopy (OS) by age at OS measurement quintiles in adolescent daughters and mothers in the Columbia-BCERP Study

1. **Water content in daughters by age at OS measurement and age at menarche**


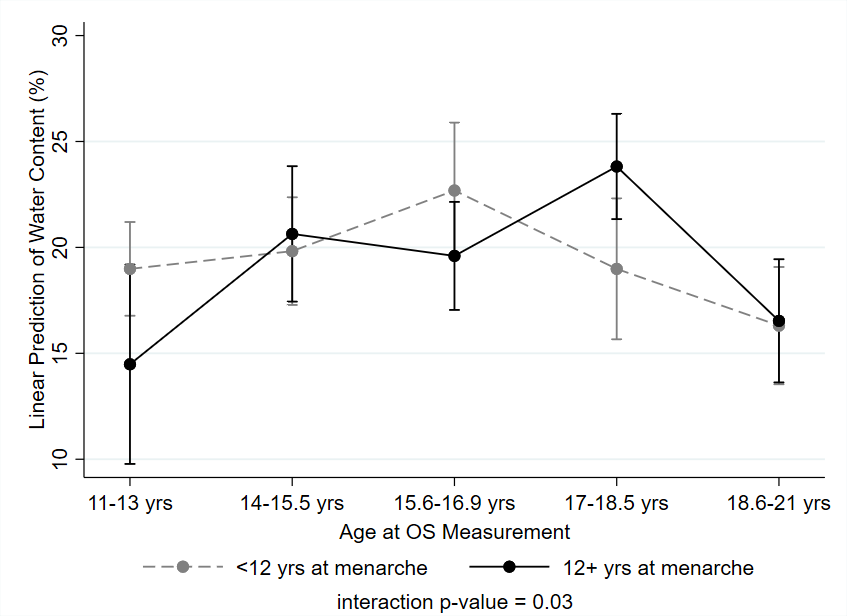


1. **Lipid content in daughters by age at OS measurement and age at menarche**


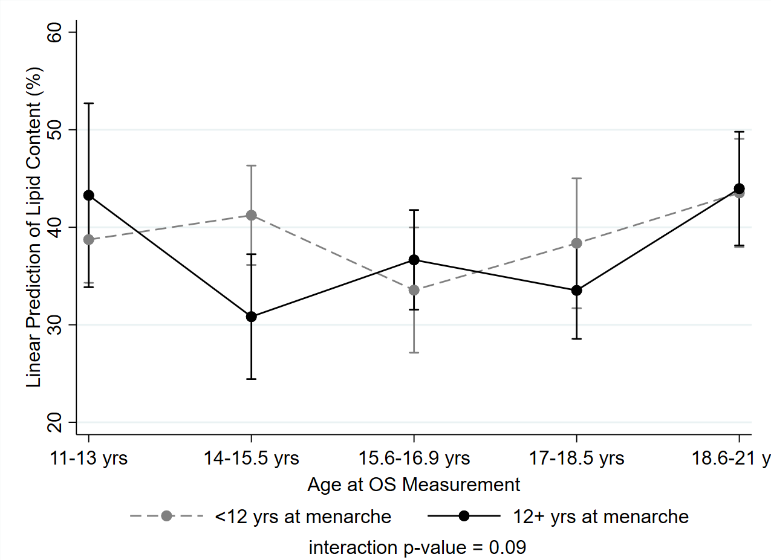


1. **Collagen content in daughters by age at OS measurement and age at menarche
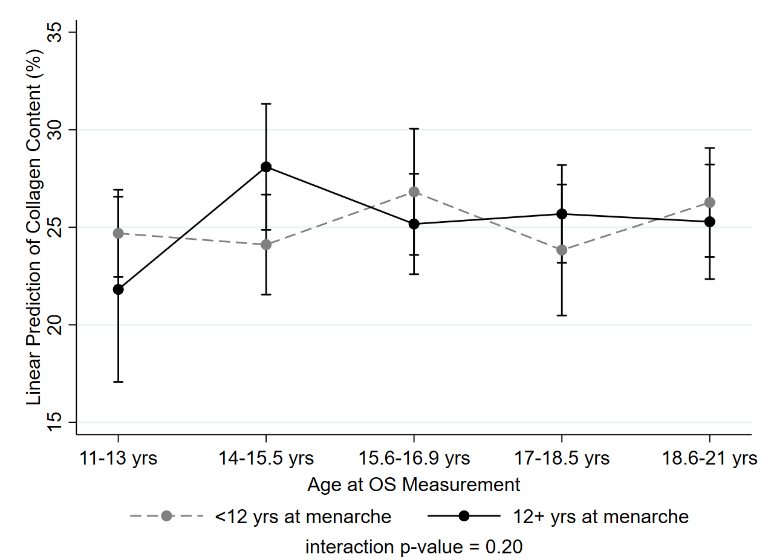
**
2. **Water content in mothers by age at OS measurement
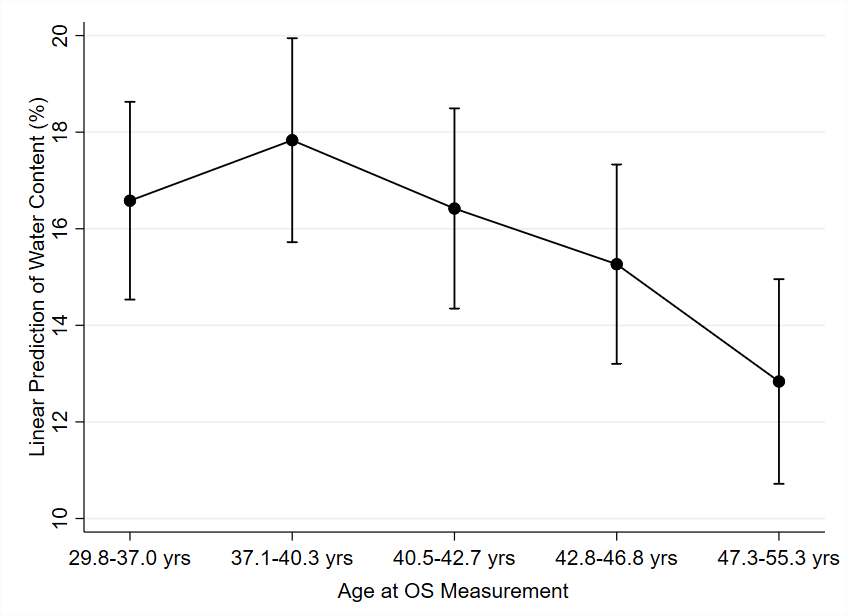
**
3. **Lipid content in mothers by age at OS measurement**
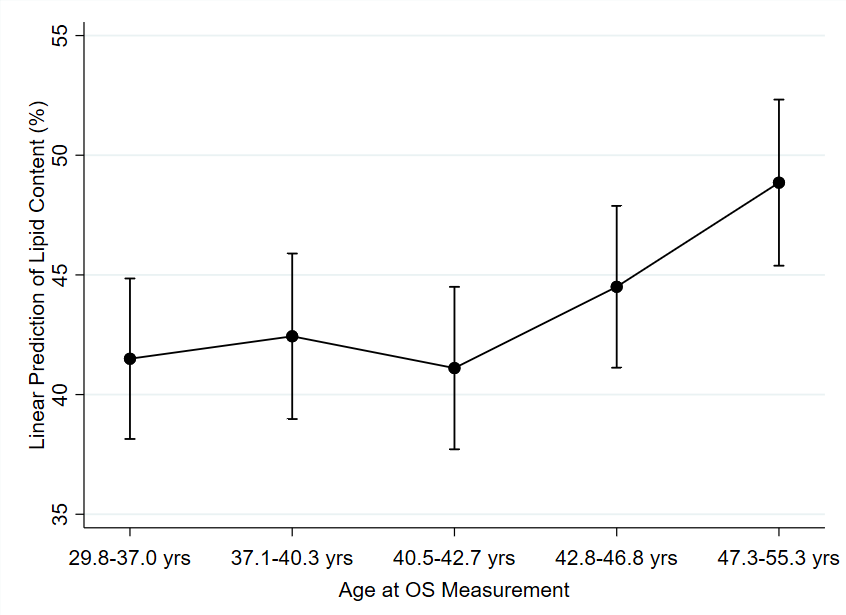

4. **Collagen content in mothers by age at OS measurement and age at menarche**

**
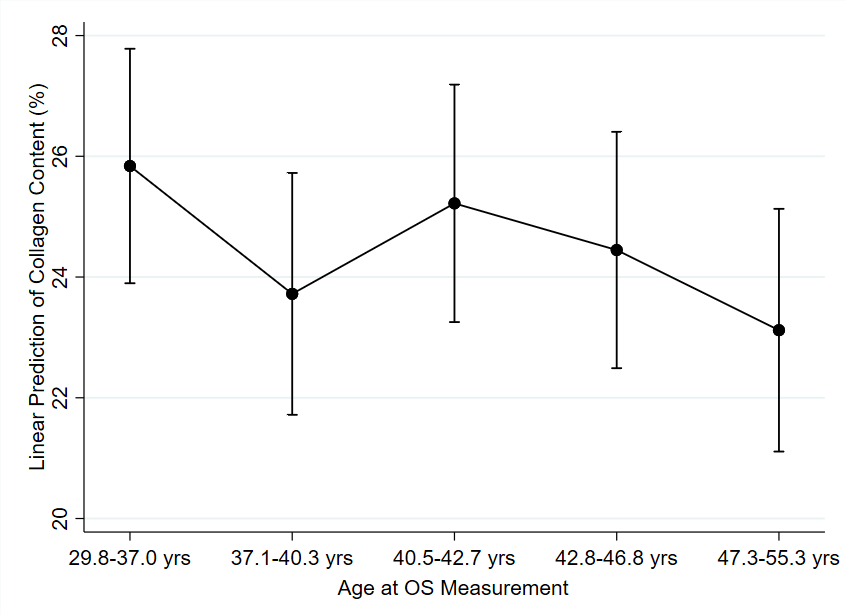
**

**Footnote:** Predicted means are adjusted for ethnicity and percent body fat at optical spectroscopy (OS) measurement. Interaction p-values in figures of daughters correspond to the Wald test statistic for the cross-product term between age at OS and menarche predicting BTC.
